# Macroporous Resin Purification of Phenolics from *Penthorum* *chinense* Leaves: Phenolic Identification, Composition Analysis, and Biological Activities

**DOI:** 10.3390/antiox15060709

**Published:** 2026-06-03

**Authors:** Qian Lai, Junlin Deng, Manyou Yu, Lu Gan, Yongqing Zhu, Chen Xia, Youmin Ying, Zhuoya Xiang

**Affiliations:** 1 Institute of Agro-Products Processing Science and Technology (Institute of Food Nutrition and Health), Sichuan Academy of Agricultural Sciences, 60 Shizishan Road, Chengdu 610066, China; 2 Gulin County Agricultural Technology Extension and Service Center, Gulin 646500, China; 3 College of Pharmaceutical Science, Zhejiang University of Technology, Hangzhou 310014, China

**Keywords:** *Penthorum chinense*, purification, phenolic identification, composition analysis, biological activities

## Abstract

In this study, 20% ethanol elution fraction(PC-20), 40% ethanol elution fraction(PC-40), 60% ethanol elution fraction(PC-60), and 80% ethanol elution fraction (PC-80)of *Penthorum chinense* polyphenols were obtained using AB-8 macroporous resin . Their in vitro bioactivities were compared to explore potential applications. A comprehensive phytochemical analysis identified 85 compounds, including 16 phenolic acids, 36 flavonoids, 24 hydrolyzed tannins, 7 anthocyanins, and 2 others. The results showed clear ethanol concentration-dependent variations in both compound composition and bioactivity. PC-20 had the highest levels of total polyphenols (418.45 mg/g), proanthocyanidins (84.95 mg/g), and tannins (10.61 mg/g), and also showed the best antioxidant capacity. PC-40 contained the most flavonoids (227.55 mg/g). PC-60 gave the strongest α-glucosidase inhibition (IC_50_ = 0.79 µg/mL), while PC-20 was most effective against pancreatic lipase (IC_50_ = 101.06 µg/mL) and also significantly activated the enzymes ADH and ALDH. Overall, PC-20 appears more suitable for applications aimed at antioxidant, anti-obesity, or liver-protective effects, whereas PC-60 is more promising for blood glucose control. This work provides a practical basis for selecting different ethanol fractions of *P. chinense* polyphenols according to specific functional needs.

## 1. Introduction

*Penthorum chinense* Pursh (known as “Gan-Huang-Cao” or “Che-Gen-Cai” in Chinese; family Penthoraceae) is a perennial herb widely used in traditional Chinese medicine for its hepatoprotective effects against conditions such as liver edema, infectious hepatitis, and chemical-induced liver injuries [1,2]. The plant is mainly found in Gulin County in southwestern China, an area inhabited by the Miao ethnic group and recognized as the geo-authentic production region for *P. chinense*. Because of its therapeutic efficacy, it is locally called the “immortal herb” and is also eaten as a vegetable [3].

Recent studies have shown that *P. chinense* has a broad range of biological activities, including antioxidant, lipid-regulatory, and anti-cancer effects [4,5,6]. These benefits are largely due to its rich and diverse secondary metabolites, especially phenolic compounds such as flavonoids, phenolic acids, and their derivatives [1]. To date, more than 100 compounds have been isolated from *P. chinense*, with flavonoids being the major group [4,7,8,9]. As potent natural antioxidants, these phenolic compounds not only contribute to the plant’s own defense system but also show promise in alleviating oxidative stress—a key factor in the development of various chronic diseases in humans [2].

In recent years, the leaves of *P. chinense* have attracted increasing scientific interest as a valuable source of these bioactive phenolics [10]. However, full use of their potential is often limited by the inherent complexity of the crude extracts. Such extracts typically contain many interfering compounds—sugars, proteins, and pigments—that can obscure accurate compositional analysis, reduce biological efficacy per unit mass, and make standardization difficult for practical applications [11,12]. Thus, efficient and scalable purification techniques are needed to enable precise characterization and better use of these natural antioxidants.

Among various purification strategies, macroporous resins have become an attractive and sustainable technology for enriching bioactive compounds from plant materials [13]. Their operation is based on adsorption and desorption, offering advantages such as high adsorption capacity, good selectivity, ease of use, and cost-effectiveness [14,15]. Moreover, the process is environmentally friendly, using mainly water and food-grade ethanol as solvents, which makes it suitable for applications in the food, pharmaceutical, and nutraceutical industries [16,17]. Recent studies have successfully applied macroporous resins to purify phenolic compounds from several botanical sources, including *C. polyodonta* flowers [18], *Citrus aurantium* L. [19], and *Vernonia patula* (Dryand.) Merr. [20], with clear improvements in biological activity. Nevertheless, the systematic use of macroporous resin purification for enriching phenolics specifically from *P. chinense* leaves remains largely unexplored.

In this study, we used AB-8 resin to purify the phenolics from *P. chinense* leaves, identify and quantify the compounds, and determine the total phenolics, flavonoids, proanthocyanidins, tannins, antioxidant, enzyme inhibition, ADH/ALDH activation, and cell-based hepatoprotective activities. The whole point was to see if this resin works for *P. chinense* and how the chemical profile ties to function.

## 2. Materials and Methods

### 2.1. Materials and Chemicals

*P. chinense* leaves were collected from Huangjing Town (Luzhou, China) in August 2022. The fresh PC leaves were frozen at −80 °C for 12 h and then freeze-dried using a freeze-dryer (EYELA FDU-2110, Tokyo Rikakikai Co., Ltd., Tokyo, Japan) at −80 °C and for 48 h, ground into 60-mesh particles, and stored at −20 °C for testing.

AB-8 macroporous adsorption resin (500 g, BR grade) was obtained from Shanghai Amole Bio-Technology Co., Ltd. (Shanghai, China). Metaphosphoric acid, gallic acid, protocatechuic acid, catechin, epicatechin, rutin, isoquercitrin, kaempferol-3-*O*-rutinoside, astragalin, afzelin, pinocembrin-7-*O*-glucoside, quercetin, kaempferol, PGHG, pinocembrin, thonningianin A, pepsin, trypsin, acetaldehyde dehydrogenase, β-nicotinamide adenine dinucleotide (NAD^+^), α-glucosidase, quinocidine dimethylacrylic acid, ABTS (2,2′-azino-bis(3-ethylbenzothiazoline-6-sulfonic acid)) diammonium salt, lipase (from porcine pancreas), Triton X-100, sodium taurocholate, tris(hydroxymethyl)aminomethane (Tris), 2,2-diphenyl-1-picrylhydrazyl (DPPH), 4-nitrophenyl laurate, and alcohol dehydrogenase were purchased from Shanghai Yuanye Bio-Technology Co., Ltd. (Shanghai, China). Ethanol, methanol, Folin–Ciocalteu reagent, sodium carbonate, aluminum nitrate, sodium hydroxide, sodium acetate, hydrochloric acid, sulfuric acid, glacial acetic acid, isopropanol, disodium hydrogen phosphate, sodium dihydrogen phosphate, sodium pyrophosphate, gelatin, sodium tungstate, phosphomolybdic acid, and phosphoric acid were purchased from Chengdu Kelong Chemical Reagent Factory (Chengdu, China). Total protein (TP), aspartate aminotransferase (GOT), alanine aminotransferase (GPT), and lactate dehydrogenase (LDH) assay kits were purchased from Nanjing Jiancheng Bioengineering Institute (Nanjing, China).

### 2.2. Sample Extraction and Enrichment of Phenolics

An amount of 100 g *P. chinense* leaf powder was mixed with 1000 mL of 70% ethanol. The mixture was sonicated for 30 min at 40 °C, and the extract was separated by centrifugation (7000 rpm, 10 min). The supernatant was collected and the extraction procedure was repeated twice. Subsequently, the supernatant was concentrated using Hei-VAP Advantage rotary evaporators, employing reduced pressure at 40 °C. The concentrated solution was then freeze-dried for the crude *P. chinense* leaf extract (PC).

Based on our previously described methods [18], enrichment experiments were conducted using four glass columns (5 cm × 60 cm), which were packed with AB-8 resins. The PC was dissolved in 200 mL of distilled water and prepared at a concentration of 25 mg/mL. This PC solution was adsorbed in the resins for 12 h to reach adsorption equilibrium. The column was subjected to sequential elution using different ethanol concentrations: 800 mL of distilled water, followed by 20% (*v*/*v*) ethanol (PC-20), 40% (*v*/*v*) ethanol (PC-40), 60% (*v*/*v*) ethanol (PC-60), and 80% (*v*/*v*) ethanol (PC-80). The elution was carried out at a constant flow rate of 0.5 mL/min and a temperature of 24 °C.

The PC-20, PC-40, PC-60 and PC-80 were collected individually and were concentrated using Hei-VAP Advantage rotary evaporators, employing reduced pressure at 40 °C. The sample was frozen at −80 °C for 12 h and then freeze-dried using a freeze-dryer (EYELA FDU-2110, Tokyo Rikakikai Co., Ltd., Japan) at −80 °C for 48 h to obtain dry powder. The dried fractions were weighed, stored at room temperature in a drying cabinet, and reserved for subsequent use.

### 2.3. Composition Analysis

#### 2.3.1. Identification of Phenolic Compounds Using UPLC-Q-TOF-MS/MS

Phenolic compounds in PC were identified and detected using a UPLC system coupled with a PDA detector and a Waters Xevo G2-XS QTOF mass spectrometer (ESI source), following our previously described method [21]. The separation was performed on a Waters BEH C_18_ column (2.1 mm × 100 mm, 1.7 μm).

For HPLC-DAD analysis, eluent A was 0.1% formic acid in water, and eluent B was acetonitrile. The gradient program was: 0–5 min (5–10% B), 5–8 min (10–20% B), 8–14 min (20–40% B), 14–18 min (40–80% B), and 18–20 min (80–100% B). The injection volume was 1 µL, and the flow rate was 0.3 mL/min. PDA spectra were recorded at 280 nm and 350 nm for phenolic compounds.

MS analysis was performed in negative ionization (NI) mode. The conditions were: capillary voltage 2.5 kV, desolvation gas flow 600 L/h, cone gas flow 50 L/h, desolvation temperature 250 °C, source temperature 120 °C, and scan range *m*/*z* 50–1500. The UV chromatogram of the crude extract is shown in Figure S1. MALDI-NLynx software (version 4.1, Waters Corporation, Milford, MA, USA) was used for data processing and compound identification. Putative identities were assigned by comparing the observed MS/MS fragmentation patterns with those reported in the literature on *Penthorum chinense* and related plants.

#### 2.3.2. Total Phenolic Content (TPC)

The TPC was determined using Folin–Ciocalteu method [18]. A total of 20 μL of the diluted extract solution was mixed with 20 μL Folin–Ciocalteu and react for 5 min. Then, 5% Na_2_CO_3_ (160 μL) was added and mixed evenly. The reaction was carried out at room temperature in the dark for 1 h, and the OD value of the sample was detected at 765 nm. The standard curve of gallic acid was *y* = 0.0072*x* + 0.001 (*R*^2^ = 0.9991), and the results were expressed as mg gallic acid equivalent (GAE)/g dry weight (DW).

#### 2.3.3. Total Flavonoid Content (TFC)

The TFC was determined using the NaNO_2_-Al(NO_3_)_3_-NaOH colorimetric method [18]. Briefly, 20 μL of diluted extract was mixed with 15 μL of 5% NaNO_2_ and allowed to react for 6 min at room temperature. Then, 15 μL of 10% AlCl_3_·6H_2_O was added, followed by shaking and a 5 min incubation. After adding 100 μL of 1 mol/L NaOH, the absorbance was measured at 510 nm. A rutin standard curve (*y* = 0.0017*x* − 0.0021, *R*^2^ = 0.9994) was used for quantification. Results are expressed as mg rutin equivalent (RE)/g dry weight (DW).

#### 2.3.4. Total Proanthocyanidin Content (TPAC)

The TPAC was determined by the method of Tong et al. [22] with slight modifications. Briefly, 1 mL of extract was mixed with 3 mL of 40 g/L vanillin-methanol solution and 1.5 mL of concentrated hydrochloric acid. The mixture was incubated at 45 °C in the dark for 30 min. Absorbance was then measured at 500 nm. A catechin standard curve (*y* = 0.0032*x* + 0.0074, *R*^2^ = 0.9993) was used for quantification. Results are expressed as mg catechin equivalent (CE)/g dry weight (DW).

#### 2.3.5. Total Tannin Content (TTC)

The TTC was determined using the Li method with minor modifications [23]. Specifically, 0.5 mL of extract was sequentially mixed with 0.85 mL of 70% ethanol, 0.05 mL of 60 mg/mL metaphosphoric acid solution, 12.5 mL of water, 1.25 mL of Folin-Denis reagent, and 5 mL of 1 mol/L sodium carbonate solution. The mixture was shaken vigorously, diluted to the mark with water, and then incubated in a thermostat at 39 °C for 1.5 h. The absorbance was measured at 680 nm using a spectrophotometer. The standard curve was described by the equation *y* = 0.0834*x* + 0.0514 (*R*^2^ = 0.9946). Results were expressed as milligrams of tannic acid equivalent (TAE)/g dry weight (DW).

#### 2.3.6. Analysis of Phenolic Composition

The extracts were analyzed using an Agilent LC-1290 HPLC system (Agilent, Santa Clara, CA, USA) [18]. Chromatographic separation was performed on a PFP column (4.6 × 100 mm, 2.7 μm). The mobile phase consisted of 0.1% formic acid in water (A) and acetonitrile (B). The gradient elution program was as follows: 0–10 min, 5–10% B; 10–20 min, 10–20% B; 20–27.5 min, 20–30% B; 27.5–30 min, 30–90% B; 30–32 min, 90% B. The flow rate was 0.8 mL/min, the injection volume was 2 μL, and the column temperature was maintained at 35 °C. Detection wavelengths were set at 280 nm and 350 nm. Results were expressed as micrograms of phenolics/g dry weight (DW).

### 2.4. Determination of Antioxidant Activity

#### 2.4.1. DPPH Free Radical Scavenging Ability

DPPH free radical scavenging activity was determined using the Aisha method [24]. First, 100 μL sample and 100 μL of DPPH solution were mixed and allowed to react for 30 min in the dark. The absorbance was read at 517 nm, and vitamin E was used as a positive control. The DPPH free radical scavenging activity was calculated using Formula (1):

(1) $$DPPH\;free\;radical\;scavenging\;ability(\%)=\frac{\left(A0-A1\right)}{A0}\times 100$$

where *A*0 represents the absorbance of the blank group, and *A*1 represents the absorbance of the sample group.

#### 2.4.2. ABTS^+^ Free Radical Scavenging Ability

ABTS^+^ free radical scavenging ability was determined by Awe method [25]. The 40 μL sample and 160 μL ABTS solution were mixed at room temperature and allowed to react in the dark for 5 min. The OD value was measured at 734 nm, and vitamin E was used as the positive control. The ABTS^+^ free radical scavenging ability was calculated according to the Formula (1).

#### 2.4.3. Ferric Reducing Antioxidant Power (FRAP)

Ferric reducing antioxidant power was determined by Zhu method with slight modification [26]. The30 μL sample and 265 μL FRAP working solution were reacted at 37 °C for 30 min. The OD value was measured at 593 nm and vitamin E used as the positive control.

### 2.5. Hypolipidemic Activity In Vitro

Pancreatic lipase inhibitory activity was determined by Wang method [27]. A 50 μL extract was mixed with 200 μL pancreatic lipase (5 mg/mL, 100 mM, pH = 8.2 Tris-HCl buffer). After incubation at 37 °C for 15 min, 50 μL 0.4% *p*NP laurate was added and 37 °C incubation for 45 min, the OD value was measured at 405 nm. Orlistat was used as the positive control. The pancreatic lipase inhibition activity was calculated using Formula (2).

(2) $$Pancreatic\;lipase\;inhibitory\;activity(\%)=\frac{A1-A0}{A2-A3}\times 100$$

where *A*1: sample + pancreatic lipase + *p*NP laurate; *A*0: sample + Tris-HCl buffer + *p*NP laurate; *A*2: Tris-HCl buffer + pancreatic lipase + *p*NP laurate; and *A*3: Tris-HCl buffer + Tris-HCl buffer + *p*NP laurate.

### 2.6. Anti-Hyperglycemic Activity In Vitro

Anti-hyperglycemic activity in vitro was evaluated by α-glucosidase inhibitory activity and was determined according to Cherrada method [28]. A 50 μL sample was mixed with 50 μL of α-glucosidase (1 U/mL, 0.1 mol/L pH = 6.9 PBS). The mixture was kept in 37 °C for 10 min. Then, 50 μL PNPG (5 mmol/L, 0.1 mol/L pH = 6.9 PBS) was added and kept in 37 °C for 5 min. Additionally, 50 μL Na_2_CO_3_ (1 mol/L) was added and the OD value was measured at 405 nm. Agarose was used as a positive control. The anti-hyperglycemic activity was calculated using Formula (3).

(3) $$\mathrm{\alpha -glucosidase}\;inhibitory\;activity=1-\frac{\left(A0-A1\right)}{A2-A3}\times 100$$

where *A*0: Sample + α-glucosidase + PNPG + Na_2_CO_3_; *A*1: sample + PBS + PNPG + Na_2_CO_3_; *A*2: PBS + α-glucosidase + PNPG + Na_2_CO_3_; and *A*3: PBS + PBS + PNPG + Na_2_CO_3_.

### 2.7. Alcohol Metabolizing Enzyme Activity In Vitro

#### 2.7.1. Alcohol Dehydrogenase (ADH) Activity

ADH activity was determined according to Yuan method with slight modification [29]. A 1.5 mL sodium diphosphate buffer (32 mM, pH = 8.8) was mixed with 1 mL NAD (32 mM, pH = 8.8), 0.5 mL ethanol (11.5%) and 100 μL sample, and incubated at 37 °C for 5 min. Then, 100 μL ADH was added and the absorbance was read at 340 nm every 10 s for 5 min until the increase in absorbance per minute reached a stable value. The ADH activity was calculated using the following Formula (4).

(4) $$Q(\%)=\frac{E1-E0}{E0}\times 100$$

where Q represents the activation rate of ADH or ALDH (%); *E*1 represents the enzyme activity in the sample solution (U/mg); and *E*0 represents the enzyme activity in a blank solution (U/mg).

#### 2.7.2. Acetaldehyde Dehydrogenase (ALDH) Activity

ALDH activity was determined according to Jae-Young method with slight modification [30]. A 1.5 mL PBS (pH = 8.0, 0.124 mol/L) was mixed with 0.5 mL substrate solution, 1.0 mL oxidized coenzyme I (NAD^+^, 16 mmol/L), and 0.1 mL sample. The mixture was kept at 37 °C for 5 min and added 0.1 mL ALDH to initiate the reaction. The absorbance was read at 340 nm every 10 s for 5 min until the increase in absorbance per minute reached a stable value. The ALDH activity was calculated using the following Formula (4).

### 2.8. Determination of Hepatoprotective Effect in Cells

#### 2.8.1. Cell Cultures

Human liver hepatoma cells (HepG2 cells) were obtained from iCell Bioscience Inc. (Shanghai, China). The cells were cultured in HepG2 cell complete culture medium (iCell Bioscience Inc., Shanghai, China) containing 10% FBS, streptomycin (100 IU/mL) and penicillin (100 IU/mL) at 5% CO_2_ and 37 °C [31].

#### 2.8.2. Assessment of Cell Viability

The MTT assay was used to measure the viability of cells treated with different purified fraction samples. HepG2 cells were plated in 96-well microplates at a density of 5 × 10^4^ cells/well. To determine the appropriate ethanol concentration for establishing the alcohol-induced injury model, cells were treated with various concentrations of ethanol (0, 100, 200, 400, 600, and 800 mM) for 24 h, and cell viability was assessed (Figure S2). Based on these results, 600 mM ethanol was selected because it reduced cell viability to approximately 50–60% of the control level, which is suitable for evaluating hepatoprotective effects. Therefore, for subsequent experiments, cells were first incubated for 24 h, then treated with 600 mM ethanol for 24 h to establish the alcohol-induced mode.

#### 2.8.3. LDH, ALT and AST Activity

LDH, ALT and AST activity in HepG2 cells was measured using an assay kit. To measure LDH activity, HepG2 cells were plated in 96-well plates at 5 × 10^4^ cells/mL. After incubation for 24 h, the cells were treated with 600 mM ethanol for 24 h, and then, followed by incubation with the samples at 1 μg/mL for 24 h. The cells were centrifuged at 350× *g* for 5 min, after which 0.01 mL of the supernatant was transferred to a new 96-well plate. Subsequently, 0.1 mL of the LDH reaction mixture was added, and the LDH activity was assessed using a microplate reader at 440 nm. LDH activity was measured in triplicate.

The AST and ALT level was determined using a commercially available assay kit. Cells (0.1 × 10^7^ cells/mL) were homogenized in 200 μL cold assay buffers and centrifuged 4× *g* for 10 min), and 25 μL of the collected supernatant was added to 50 μL of the reaction mixture in the AST kit and ALT kit. The AST and ALT level was assessed at 510 nm, and the activity was presented as U/L.

### 2.9. Statistical Analysis

All experiments were performed with three biological replicates unless otherwise specified. The data obtained were subjected to one-way analysis of variance (ANOVA) followed by Duncan’s multiple range test using SPSS 17.0 software (version 17.0, SPSS Inc., Chicago, IL, USA), with a significance level set at *p* < 0.05.

## 3. Results

### 3.1. Identification of Phenolic Compounds in P. chinense Leaves

The identification results of some compounds detected are shown in Table 1. A total of 85 compounds were identified or preliminarily characterized in *P. chinense* leaves, mainly divided into five categories: phenolic acids (16), flavonoids (36), hydrolyzed tannins (24), procyanidins (7), and others (2). The detailed analysis of the compound was as follows.

#### 3.1.1. Phenolic Acid Compounds

Sixteen phenolic acid compounds were identified from *P. chinense* leaves. The mass spectrometry of phenolic acid compounds was relatively simple, mainly due to the loss of ions such as CO_2_ (*m*/*z* 44), CO (*m*/*z* 28), HCOO (*m*/*z* 45), and H_2_O (*m*/*z* 18), specifically, as follows.

Compounds **1** and **4** show deprotonated molecule [M-H]^−^ at *m*/*z* 355.0327 (C_14_H_12_O_11_). And *m*/*z* 337 was obtained by ion rearrangement after deprotonated molecule [M-H]^−^ losing one unit of H_2_O. On this basis, one unit of CO_2_ was successively lost, producing fragment ions at *m*/*z* 293 and *m*/*z* 249, respectively. In addition, the ion *m*/*z* 205 was producing due to *m*/*z* 249 losing one unit of COCH_2_. Through literature review [32], compound **1** was tentatively identified as chebulic acid, and compound **4** was tentatively identified as a chebulic acid isomer.

Compound **5** shows the deprotonated molecule [M-H]^−^ at *m*/*z* 169.0119 (C_7_H_6_O_5_). The MS^2^ spectrum of this ion displayed a peak at *m*/*z* 125, resulting from the loss of one unit of HCOO. Through literature review [33], compound 5 was tentatively identified as gallic acid.

Compounds **10** and **13** show the deprotonated molecule [M-H]^−^ at *m*/*z* 385 (C_17_H_22_O_10_). The MS^2^ fragment ions were observed at *m*/*z* 223, *m*/*z* 205, *m*/*z* 190, *m*/*z* 175, and *m*/*z* 119. And the MS^2^ spectrum of this ion displayed a peak at *m*/*z* 223, resulting from the loss of one unit of Glu residue, on this basis, by losing again a unit of H_2_O, producing fragment ions at *m*/*z* 205. Through literature review [34], compounds **10** and **13** were tentatively identified as sinapoylglucoside, respectively.

Compound **12** shows deprotonated molecule [M-H]^−^ at *m*/*z* 300.9995 (C_14_H_6_O_8_) was further tentatively assigned as ellagic acid [35], since the MS^2^ spectrum of the ion at *m*/*z* 257 and *m*/*z* 229 were due to losses one unit of CO_2_ and CO, respectively.

Among them, compounds **2**, **3**, **6**, **7**, **8**, **9**, and **11**, based on reported literature and characteristic ion fragments, were inferred to correspond to chemical compositions of quinic acid [33], citric acid [32], methyl gallate [36], brevifolin carboxylic acid [32,37], feruloylglucose [38], ferulic acid 4-*O*-β-D-glucopyranoside [39], and vanilloylglucose [40]. Similarly, compounds **14**, **15**, and **16** have all been tentatively identified as decarboxylated 8-5′-dehydroferulic acid [41].

#### 3.1.2. Flavonoid Compounds

Thirty-six flavonoid compounds were identified from *P. chinense* leaves, most of which produce various fragment ions through the cleavage and rearrangement of the A and B rings, mainly due to the loss of rutinoside residues, gallic acid residues, loss of CHO (*m*/*z* 29), CO (*m*/*z* 28), and *m*/*z* 106 ion, specifically, as follows.

Compound **21** shows the deprotonated molecule [M-H]^−^ at *m*/*z* 289.0689 (C_15_H_14_O_6_) and was further tentatively assigned as (-)-epicatechin [32,37], since the MS^2^ spectrum of the ion at *m*/*z* 245, due to cracking the A ring. Furthermore, the MS^2^ spectrum of this ion displayed fragment ions at *m*/*z* 123 and *m*/*z* 109, resulting from *m*/*z* 245 the loss of one unit of *m*/*z* 122 and CH_2_, respectively. Compound **22** show the deprotonated molecule [M-H]^−^ at *m*/*z* 593.1497(C_30_H_26_O_13_), and the loss of rhamnoside residue leads to the kaempferol 3-glucoside ion at *m*/*z* 447. Based on this, the MS^2^ spectrum of this ion displayed fragment ions at *m*/*z* 285 and *m*/*z* 255, resulting from the loss of one unit of Glu and CO, respectively. Through a literature review [42], compound **22** was tentatively identified as kaempferol-3-*O*-glucoside-7-*O*-rhamnoside.

Among them, compounds **25**, **29**, **36**, and **43**, based on reported literature and characteristic ion fragments, were inferred to correspond to chemical compositions of kaempferol-*O*-rutinoside [43], kaempferol-3-*O*-rutinoside [32], kaempferol-3-*O*-rhamnoside [32], and kaempferol [32]. Similarly, compounds **34** and **35** have all been tentatively identified as kaempferol-3-*O*-pentoside isome [32].

Compound **24** shows the deprotonated molecule [M-H]^−^ at *m*/*z* 609.1461 (C_27_H_30_O_16_), and the loss of the rutinoside residue leads to the quercetin ion at *m*/*z* 301. Based on this, the B ring loses one unit of CHO through cracking, producing ions of *m*/*z* 271 or *m*/*z* 273, in addition producing ions of *m*/*z* 243 and *m*/*z* 255, respectively. Through literature review [43], compound 24 was tentatively identified as quercetin-3-*O*-rutinoside.

Among them, compounds **20**, **27**, **28**, **30**, **31**, and **32**, based on reported literature and characteristic ion fragments, were inferred to correspond to chemical compositions of quercetin-3-*O*-pentoside-7-*O*-glucoside [44], quercetin-3-*O*-glucoside [32], quercetin-3-*O*-arabinoside [32], quercetin-3-*O*-neohesperidoside [45], quercetin-3-*O*-xyloside [32], and quercetin-3-*O*-rhamnoside [32]. Similarly, compounds **17** and **18** have all been tentatively identified as quercetin-dihexoside isomers [32,37].

Compound **26** shows the deprotonated molecule [M-H]^−^ at *m*/*z* 441.082 (C_22_H_18_O_10_), and the loss of gallic acid residue (*m*/*z* 169) leads to the ion at *m*/*z* 289, while also producing an ion of *m*/*z* 271. On this basis, the B ring can be cracked and rearranged to produce an ion of *m*/*z* 125. Through a literature review [46], compound **26** was tentatively identified as (-)-epicatechin gallate.

Compound **38** shows the deprotonated molecule [M-H]^−^ at *m*/*z* 301.0351 (C_15_H_10_O_7_), which was further assigned as quercetin [5,32], since the MS^2^ spectrum of the ion at *m*/*z* 151 and *m*/*z* 179 was due to the cleavage of the 2,4 bonds and the 2,3 bonds in the B ring, respectively. Compound **38** was in full agreement with quercetin.

Compound **39** shows the deprotonated molecule [M-H]^−^ at *m*/*z* 417.1170 (C_15_H_10_O_7_), which was further tentatively assigned as pinocembrin-7-*O*-glucoside [32], and the loss of Glu leading to the ion at *m*/*z* 255. On this basis, the RDA cracking produces ions with *m*/*z* 151.

Compound **40** shows the deprotonated molecule [M-H]^−^ at *m*/*z* 255.0677 (C_15_H_12_O_4_), which was further tentatively assigned as pinocembrin [32], since the MS^2^ spectrum of the ion at *m*/*z* 213 was due to the loss of the COCH_2_ in the B ring.

Compound **41** shows the deprotonated molecule [M-H]^−^ at *m*/*z* 419.1353 (C_21_H_24_O_9_), and the loss of Glu leading to the ion at *m*/*z* 257. On this basis, the loss of CO_2_ leading to the ion at *m*/*z* 213, and the loss of *m*/*z* 132 leading to the ion at *m*/*z* 125. Through a literature review [32,35], compound **41** was tentatively identified as 2′,4′,6′-trihydroydi-hydrochalcon-4′-*O*-glucoside.

Compounds **50** and **51** show the deprotonated molecule [M-H]^−^ at *m*/*z* 871.1351 (C_42_H_32_O_21_), and the loss of ellagic acid residue (*m*/*z* 301), leading to the ion at *m*/*z* 569 and *m*/*z* 275. On this basis, the loss of CO_2_ and gallic acid residue led to the ion at *m*/*z* 255, and the loss of *m*/*z* 132 led to the ion at *m*/*z* 125. Through a literature review, compounds **50** and **51** were tentatively identified as pinocembrin-7-*O*-(3″-*O*-galloy-4″,6″-(S)-hexahydroxydiphenoyl)-β-D-glucoside [5,32] and pinocembrin-7-*O*-(3″-*O*-galloy-4″,6″-(R)-hexahydroxydiphenoyl)-β-D-glucoside [32,37], respectively.

Among them, compounds **42**, **44**, and **45**, based on reported literature and characteristic ion fragments, were inferred to correspond to chemical compositions of pinocembrin-*O*-galloylglucoside [32,37]. Similarly, compounds **47** and **49** have been tentatively identified as pinocembrin-7-*O*-(4″,6″-(S)-hexahydroxydiphenoyl)-β-glucoside and pinocembrin-7-*O*-(4″,6″-(R)-hexahydroxydiphenoyl)-β-glucoside, respectively [32,37].

Compound **52** shows the deprotonated molecule [M-H]^−^ at *m*/*z* 873.1490 (C_42_H_34_O_21_), and the loss of ellagic acid residue (*m*/*z* 301), leading to the ion at *m*/*z* 571 and *m*/*z* 275. On this basis, the loss of Glu and gallic acid residue led to the ion at *m*/*z* 257. Through a literature review [5,32], compound **52** was tentatively identified as thonningianin A.

Among them, compounds **19**, **23**, **33**, **37**, **46**, and **48**, based on reported literature and characteristic ion fragments, were inferred to correspond to chemical compositions of (+)-catechin [47], quercetin-3-*O*-glucuronide [48], isorhamnetin 3,7-*O*-diglucoside [49], luteolin [32], 2′,4′,6′-trihydroydi-hydrochalcon-*O*-glucoside 2′,6′-dihydroydihydrochalcone-4′-*O*-(4″,6″-hexahydroxydiphenoyl)-β-glucoside [35], and apigenin [32].

#### 3.1.3. Hydrolyzed Tannin Compounds

Compound **76** shows the deprotonated molecule [M-H]^−^ at *m*/*z* 631.0937 (C_27_H_20_O_18_), and the loss of ellagic acid residue (*m*/*z* 301), leading to the ion at *m*/*z* 329. And the *m*/*z* 301 loss of CO_2_ and CO led to the ion at *m*/*z* 229. Through a literature review [32], compound **76** was tentatively identified as 2,6-dihydroxyacetophenone-4-*O*-HHDP-glucose.

Among them, compounds **53**, **56**, **58**, and **59**, based on reported literature and characteristic ion fragments, were inferred to have corresponded to chemical compositions of galloylglucose [50]. Compounds **54**, **55**, and **57** were tentatively identified as HHDP-glucose [51], compounds **60**, **62**, **63**, **65**, and **68** were tentatively identified as digalloylglucose [52], compounds **61**, **72**, and **73** were tentatively identified as tetragalloylglucose [53], compounds **64** and **66** were tentatively identified as galloyl-HHDP-glucose isomers [54], compounds **67**, **69**, and **70** were tentatively identified as trigalloyl-glucose isomers [54], and compounds **74** and **75** were tentatively identified as pentagalloylglucose [55]. In addition, compound **71** was tentatively identified as trigalloyl-HHDP-glucose [56].

#### 3.1.4. Proanthocyanin Compounds

Compound **77** shows the deprotonated molecule [M-H]^−^ at *m*/*z* 577.1349 (C_30_H_26_O_16_), since the MS^2^ spectrum of the ion at *m*/*z* 563 was due to cracking the B ring. Furthermore, the loss of phloroglucinol residue and ion rearrangement led to the ion at *m*/*z* 451. In addition, the deprotonated molecule [M-H]^−^ was cleaved by RDA to produce an ion of *m*/*z* 425, and then lost a unit of H_2_O to produce an ion of *m*/*z* 407. And the loss of catechin residue led to the ion at *m*/*z* 289. On this basis, the cleavage of the 3,5 bonds in the B ring led to the ion at *m*/*z* 245. Through a literature review [53], compound **77** was tentatively identified as procyanidin B1.

Among them, compounds **78** and **80**, based on reported literature and characteristic ion fragments, were inferred to have corresponded to chemical compositions of procyanidin B3 and procyanidin B4, respectively [57].

Compound **79, 81, 82,** and **83** with deprotonated molecule [M-H]^−^ at *m*/*z* 729.1467 (C_37_H_30_O_16_) showed the loss of a gallic acid unit (*m*/*z* 169), leading to the peak at *m*/*z* 577, and was cleaved by RDA to produce an ion of *m*/*z* 425, and then lost a unit of H_2_O to produce an ion of *m*/*z* 407. In addition, due to successive losses of H_2_O and catechin residue, peaks were shown at *m*/*z* 559 and *m*/*z* 289, respectively, being tentatively identified as (epi)catechin-(epi)catechin-3-*O*-gallate [58].

#### 3.1.5. Others

Among them, compounds **84** and **85**, based on reported literature and characteristic ion fragments, were inferred to have corresponded to chemical compositions of (4′E)-2,3′-dihydroxy-3-methoxy-6′-methanone-benzophenone-4-*O*-β-D-glucopyranoside [32,59] and (4′E)-2,4-dihydroxy-3-methoxy-6′-methanone-benzophenone-3′-*O*-β-D-glucopyranoside [32,59].

### 3.2. Total Phenolic, Flavonoid, Proanthocyanidin and Tannin Contents

The elution yields of different ethanol fractions on AB-8 macroporous resin varied considerably. PC-60 gave the highest yield, accounting for 41.17% of the total, followed by PC-40 (25.63%), PC-20 (19.74%), and PC-80 (13.45%) (Table 2).

The four fractions also differed markedly in TPC, TFC, TAPC, and TTC. PC-20 had the highest TPC (418.45 mg GAE/g DW), TAPC (84.95 mg CE/g DW), and TTC (10.61 mg TAE/g DW). In contrast, PC-40 showed the highest TFC (227.55 mg RE/g DW). PC-80 displayed significantly higher levels of TPC, TAPC, and TFC than PC-60.

### 3.3. Quantification of Phenolic Compounds

A total of 15 phenolic compounds were identified, including gallic acid, protocatechuic acid, catechin, epicatechin, rutin, isoquercitrin, kaempferol 3-*O*-rutinoside, astragalin, afzelin, pinocembrin 7-*O*-β-D-glucoside, quercetin, kaempferol, PGHG, ponocembrin, and thonningianin A (Table 3).

Catechin, epicatechin, protocatechuic acid, and gallic acid were enriched in PC-20. The content of catechin in PC-20 was 13.52 times that in the crude extract (PC). Flavonoids such as rutin, isoquercitrin, kaempferol 3-*O*-rutinoside, and astragalin were mainly enriched in PC-40. Further analysis revealed that 60% ethanol effectively eluted afzelin, pinocembrin-7-*O*-glucoside, and quercetin, with quercetin reaching its peak concentration in PC-60. PGHG and thonningianin A, as key bioactive constituents of *P. chinense*, were predominantly enriched in PC-60. At 80% ethanol, only four monomeric phenolic compounds were desorbed.

### 3.4. Antioxidant Activity

**Figure 1:**
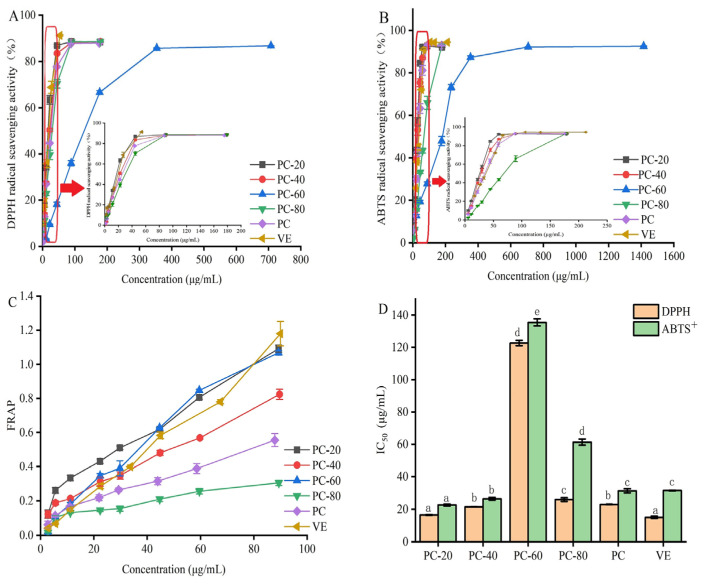
DPPH (**A**) and ABTS^+^ (**B**) free radical scavenging ability, FRAP (**C**) and IC_50_ (**D**) value of *P. chinense* different eluate fractions. Different lowercase letters indicate significant differences between the columns of the same color.

In Figure 1, based on the DPPH, ABTS, and FRAP assays, PC-20 showed the strongest overall antioxidant capacity among the *P. chinense* eluate fractions. It gave the lowest IC_50_ values in both the DPPH and ABTS assays, second only to VE, indicating superior radical scavenging activity. In the FRAP assay, PC-20 had the highest iron reduction ability at low concentrations (0–29 μg/mL), whereas PC-60 performed better at moderate concentrations (29–59 μg/mL). Still, taking all three assays together, PC-20 consistently outperformed the other fractions.

### 3.5. Anti-Hyperglycemic Activity

**Figure 2:**
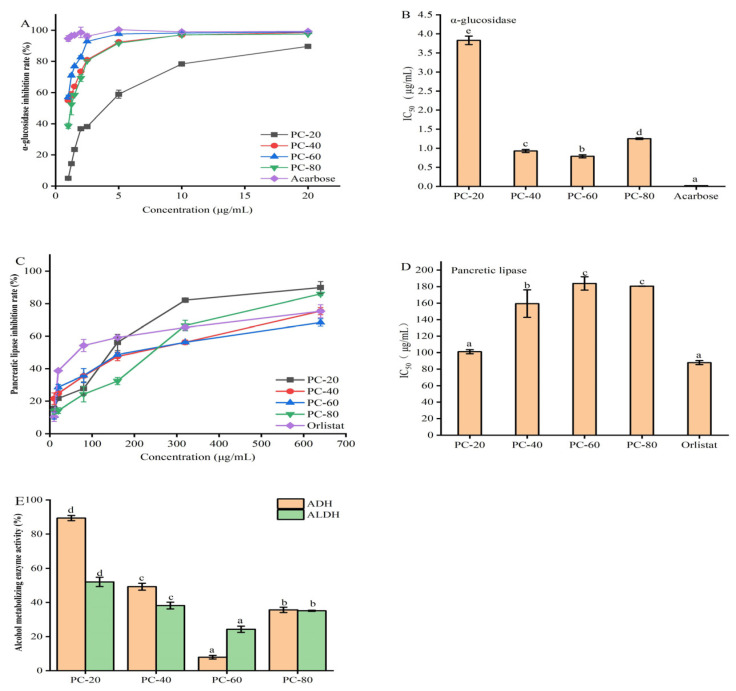
In vitro anti-hyperglycemic activity (**A**), IC_50_ values of α-glucosidase inhibition (**B**), hypolipidemic activity in vitro (**C**), IC_50_ values of pancreatic lipase inhibition (**D**), alcohol metabolizing enzyme activity (**E**) of *P. chinense* different eluate fractions. Different lowercase letters indicate significant differences between the columns of the same color.

The α-glucosidase inhibitory activities of four purified fractions from *P. chinense* are shown in Figure 2A,B. PC-60 exhibited the lowest IC_50_ value (0.79 ± 0.04 μg/mL), followed by PC-40 (0.93 ± 0.04 μg/mL), PC-80 (1.25 ± 0.02 μg/mL), and PC-20 (3.83 ± 0.11 μg/mL). This indicates that PC-60 had the strongest inhibitory activity against α-glucosidase compared to the other three purified fractions. According to Table 3, PC-60 enriched abundant kaempferol, PGHG, and thonningianin A.

### 3.6. Hypolipidemic Activity

The inhibitory effects of four purified fractions from *P. chinense* on pancreatic lipase were evaluated (Figure 2C,D). PC-20 exhibited the strongest inhibitory effect (IC_50_ = 101.06 μg/mL), followed by PC-40 (IC_50_ = 159.41 μg/mL), PC-80 (IC_50_ = 180.59 μg/mL), and PC-60 (IC_50_ = 183.89 μg/mL). Correlation analysis revealed negative correlations between IC_50_ values for lipase inhibition and total phenolic content (TPC, *R* = −0.791), total flavonoid content (TFC, *R* = −0.705), total proanthocyanidin content (TAPC, *R* = −0.874), and total tannin content (TTC, *R* = −0.670). As shown in Table 3, PC-20 contained the highest content of catechin, epicatechin, TPC, TAPC, and TTC.

### 3.7. ADH and ALDH Activities

The effects of PC-20, PC-40, PC-60, and PC-80 on ADH and ALDH activities are shown in Figure 2E. At sample concentrations of 25 μg/mL and 12.50 μg/mL, all four fractions promoted ADH and ALDH activities. The promoting effect ranked as follows: PC-20 > PC-40 > PC-80 > PC-60. Notably, PC-20 exhibited the strongest activation ability, increasing ADH activity by 89% and ALDH activity by 52%.

### 3.8. Cytoprotective Effect Against Alcohol-Induced Toxicity

**Figure 3:**
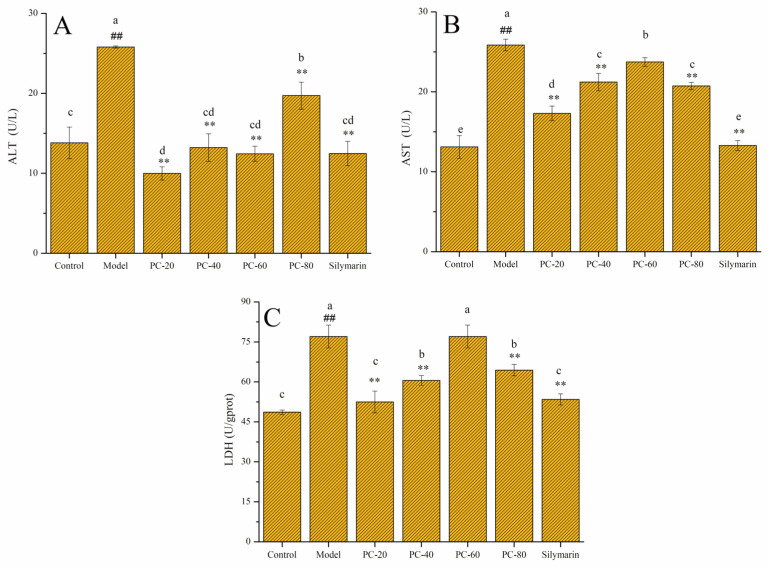
The ALT activity (**A**), AST activity (**B**) and LDH activity (**C**) of HepG2 cells pretreated with *P. chinense* different eluate fractions. Different lowercase letters indicate significant differences between the columns of the same color. Compared with the Control group, ## *p* < 0.01; compared with the Model group, ** *p* < 0.01.

The cytoprotective effects of PC-20, PC-40, PC-60, and PC-80 were examined in ethanol-stimulated HepG2 cells using the MTT assay (Figure S3). A significant increase in cell viability was observed in cells exposed to EtOH for 24 h compared to the Model group. All four fractions at 1 μg/mL showed protective effects against alcohol-induced cytotoxicity. However, cell viability decreased with increasing concentration. Therefore, 1 μg/mL was selected as the optimal concentration for subsequent experiments. The activities of ALT, AST, and LDH in HepG2 cells pretreated with the four fractions were measured (Figure 3). ALT activity (Figure 3A): PC-20 (9.99 U/L), PC-40 (13.23 U/L), PC-60 (12.45 U/L), and PC-80 (19.71 U/L) were all significantly lower than the Model group (25.80 U/L, *p* < 0.01). AST activity (Figure 3B): PC-20 (17.31 U/L), PC-40 (21.22 U/L), and PC-80 (20.73 U/L) were significantly lower than the Model group (25.86 U/L, *p* < 0.01). LDH activity (Figure 3C): PC-20 (52.47 U/gprot), PC-40 (60.52 U/gprot), and PC-80 (64.44 U/gprot) were significantly lower than the Model group (77.03 U/gprot, *p* < 0.01). Overall, PC-20 exhibited the most excellent hepatoprotective effect compared to PC-40, PC-60, and PC-80.

## 4. Discussion

The adsorption and desorption behavior of phenolic compounds from *P. chinense* leaves on AB-8 macroporous resin was consistent with their polarity and structural characteristics [47]. Similar separation patterns have been widely reported using other stationary phases; for example, D101 macroporous resin has also been shown to effectively enrich phenolic compounds from *P. chinense* [10]. Therefore, PC-20 was enriched in highly polar compounds (e.g., catechin, epicatechin, gallic acid), PC-40 in flavonoid glycosides (e.g., rutin, isoquercitrin), and PC-60 in less polar aglycones (e.g., quercetin) and specific glycosides (e.g., pinocembrin-7-*O*-glucoside). These patterns are attributable to the increasing ethanol concentration gradually disrupting hydrogen-bonding interactions and reducing solvent polarity [12]. The highest TFC in PC-40, rather than PC-20, suggests that flavonoids with moderate polarity are better eluted at slightly higher ethanol concentrations. The higher TPC, TAPC, and TFC levels in PC-80 compared to PC-60 can be explained by ethanol’s ability to effectively disrupt hydrogen-bonding interactions between flavonoids and alkaloids in *P. chinense* leaf extract, thereby promoting the release of strongly adsorbed flavonoid compounds [60].

The antioxidant activity of the four fractions was evaluated using DPPH, ABTS, and FRAP assays. The superior DPPH and ABTS radical scavenging activities of PC-20 are consistent with its highest total phenolic, proanthocyanidin, and tannin contents. Pearson correlation analysis confirmed that TPC, TFC, and TPAC were all significantly negatively correlated with ABTS (*R* = −0.939, −0.879, and −0.860, respectively, *p* < 0.01) and with DPPH (*R* = −0.812, −0.723, and −0.697, respectively, *p* < 0.01 or *p* < 0.05), indicating that these components collectively contribute to antioxidant activity (Table S1). These findings align with previous reports on *P. chinense*, where antioxidant activity was significantly positively correlated with total polyphenol content [32]. Notably, Liu et al. [10] also reported that D101 resin-enriched fractions from *P. chinense* exhibited strong DPPH and ABTS scavenging activity, further supporting our results. In contrast, PC-60 showed stronger ferric reducing power in the FRAP assay at higher concentrations, likely due to its enrichment of aglycone flavonoids (e.g., quercetin), which are known to exhibit higher reducing capacity than their glycosylated forms [61].

Regarding enzyme inhibitory activities, PC-60 exhibited the strongest α-glucosidase inhibition. This is consistent with the presence of kaempferol in PC-60, which has been reported to bind to the active site of α-glucosidase and induce conformational changes [62]. More broadly, phenolic compounds from *P. chinense* have been recognized as potential α-glucosidase inhibitors [63]. Interestingly, Pearson analysis showed that TPC and TPAC were significantly positively correlated with α-glucosidase IC_50_ (*R* = 0.596 and 0.682, respectively, *p* < 0.05), while TFC showed no significant correlation (*R* = 0.464, *p* > 0.05). These findings indicate that higher levels of total polyphenols and proanthocyanidins are associated with weaker enzyme inhibition, suggesting that the strong activity of PC-60 is likely due to specific flavonoid aglycones (e.g., kaempferol) rather than bulk phenolic components. In contrast to α-glucosidase inhibition, a different pattern was observed for pancreatic lipase inhibition. PC-20 showed the highest pancreatic lipase inhibitory activity, which correlates with its high levels of flavan-3-ols (catechin, epicatechin) and proanthocyanidins. This is in agreement with Buchholz & Melzig [64], who concluded that polyphenolic compounds, especially proanthocyanidins, act as effective pancreatic lipase inhibitors. Lachowicz et al. [65] similarly revealed that extracts of Saskatoon berry (*Amelanchier alnifolia*) displayed high pancreatic lipase inhibitory activity, which was strongly related to high polyphenol content. The strong negative correlations observed between IC_50_ values and TPC (*R* = −0.775, *p* < 0.05), TAPC (*R* = −0.853, *p* < 0.01), and TTC (*R* = −0.716, *p* < 0.05) further support that phenolic compounds, particularly proanthocyanidins and tannins, are key contributors to pancreatic lipase inhibition. These findings are consistent with Cai et al. [66], who found a strong correlation between the total phenolic content of plant extracts and enzyme inhibitory activity. Consistent with our results, Wang et al. [67] recently reported that tannins from *P. chinense* exhibited potent inhibitory activities against both α-glucosidase and pancreatic lipase.

In the alcohol metabolism assay, all four fractions promoted ADH and ALDH activities, indicating that *P. chinense* can enhance the activity of these key enzymes, thereby accelerating alcohol metabolism and exerting a liver-protective effect. The superior activation ability of PC-20 (89% for ADH and 52% for ALDH) may be related to its higher content of total polyphenols and total proanthocyanidins. The Pearson correlation analysis provides compelling statistical support for this interpretation. Both ADH and ALDH activities showed extremely strong positive correlations with TPAC (*R* = 0.905 and *R* = 0.872, respectively, *p* < 0.01) and with total polyphenol content (*R* = 0.882 and *R* = 0.862, *p* < 0.01). Consistent with this, Zhao et al. [63] reported that such compounds exert anti-alcohol effects through mechanisms including accelerating ethanol metabolism and scavenging free radicals. Accordingly, PC-20 pretreatment significantly reduced alcohol-induced elevation of ALT, AST, and LDH in HepG2 cells, indicating preserved membrane integrity and reduced oxidative damage. These hepatoprotective effects are closely associated with the strong antioxidant capacity of PC-20, consistent with its highest radical scavenging activity.

In summary, the different ethanol-eluted fractions of *P. chinense* exhibited distinct bioactivity profiles, with PC-20 being the most promising for antioxidant, pancreatic lipase inhibitory, and hepatoprotective applications, while PC-60 showed superior α-glucosidase inhibition. These findings are well supported by previously published data on *P. chinense* and related natural products.

## 5. Conclusions

This study examined how ethanol concentration affects the desorption of bioactive compounds from *P. chinense* and biological activities. Low-concentration ethanol (20%) was best for eluting total polyphenols, proanthocyanidins, and tannins, while medium concentration (40%) worked best for flavonoids. PC-20 showed the strongest antioxidant activity across DPPH, ABTS^+^, and FRAP assays, consistent with its high polyphenol content. PC-60 had the strongest α-glucosidase inhibition, suggesting anti-diabetic potential. PC-20 also showed the highest pancreatic lipase inhibition and the strongest activation of alcohol-metabolizing enzymes ADH and ALDH (potential for anti-hangover products). These findings support the targeted use of *P. chinense* in functional foods and nutraceuticals.

## Figures and Tables

**Table 1:** Characterization of phenolic compounds in *P. chinense* leaves using UPLC-Q-TOF-MS.

NO.	Rt (min)	Compound	Molecular Fomula	MS/MS
Phenolic acid				
1	0.851	chebulic acid	C_14_H_12_O_11_	355.0327, 337, 249, 205, 193
2	0.87	(iso)citric acid	C_6_H_8_O_7_	191.0151, 111, 87, 85
3	1.031	citric acid	C_6_H_8_O_7_	191.0179, 111, 87, 85
4	1.034	chebulic acid isomer	C_14_H_12_O_11_	355.0249, 337, 249, 205, 193
5	1.524	gallic acid	C_7_H_6_O_5_	169.0119, 125
6	4.538	methyl gallate	C_8_H_8_O_5_	183.0284, 124
7	4.847	brevifolin carboxylic acid	C_13_H_8_O_8_	291.0150, 247
8	5.271	feruloylglucose	C_16_H_20_O_9_	355.1060, 193, 175, 160, 132
9	5.287	ferulicacid 4-*O*-*β*-D-glucopyranoside	C_16_H_20_O_9_	355.1021, 193, 175, 160, 132
10	5.386	sinapoylglucoside	C_17_H_22_O_10_	385.1202, 223, 205, 190, 175, 119
11	6.003	vanilloylglucose	C_14_H_18_O_9_	329.0858, 209, 191, 167, 123
12	6.775	ellagic acid	C_14_H_6_O_8_	300.9995
13	8.789	sinapoylglucoside	C_17_H_22_O_10_	385.1483, 223, 193, 135
14	13.242	decarboxylated 5-8′-dehydrodiferulic acid 1	C_19_H_18_O_6_	341.1036, 326, 281, 266
15	13.603	decarboxylated 5-8′-dehydrodiferulic acid 2	C_19_H_18_O_6_	341.0998, 326, 281, 266
16	14.003	decarboxylated 5-8′-dehydrodiferulic acid 3	C_19_H_18_O_6_	341.1036, 326, 281, 266
Flavonoids				
17	4.283	quercetin-dihexoside isomers 1	C_27_H_30_O_17_	625.1044, 463,301, 300, 271, 243
18	4.559	quercetin-dihexoside isomers 2	C_27_H_30_O_17_	625.1044, 463, 301, 300, 271, 257
19	4.595	(+)-catechin	C_15_H_14_O_6_	289.0724, 245,123,109
20	5.298	quercetin 3-O-hexoside-7-*O*-rhamnoside	C_27_H_30_O_16_	609.1511, 463, 447, 301, 299, 271, 255, 151
21	5.574	(-)-epicatechin	C_15_H_14_O_6_	289.0689, 245, 123, 109
22	5.818	kaempferol-3-*O*-glucoside-7-*O*-rhamnoside	C_27_H_30_O_15_	593.1497, 447, 285, 284, 255, 227
23	6.732	quercetin-3-*O*-glucuronide	C_21_H_18_O_13_	477.0719, 315, 300, 271, 243, 149
24	6.875	quercetin-3-*O*-rutinoside	C_27_H_30_O_16_	609.1461, 301, 300, 255, 243, 151
25	7.092	kaempferol-*O*-rutinoside 1	C_27_H_30_O_15_	593.1547, 285, 284, 255, 227
26	7.115	(-)-epicatechin gallate	C_22_H_18_O_10_	441.0821, 289, 169, 125
27	7.119	quercetin-3-*O*-glucoside	C_21_H_20_O_12_	463.0819, 301, 300, 271, 255, 243, 227
28	7.379	quercetin-3-*O*-arabinoside	C_20_H_18_O_11_	433.0761, 301, 300, 271, 255, 243, 151
29	7.453	kaempferol-3-*O*-rutinoside 2	C_27_H_30_O_15_	593.1447, 285, 284, 255, 227
30	7.616	quercetin-3-*O*-neohesperidoside	C_27_H_30_O_16_	609.1511, 301, 300, 255, 243, 151
31	7.655	quercetin-3-*O*-xyloside	C_20_H_18_O_11_	433.0761, 301, 300, 271, 255, 243, 151
32	7.801	quercetin-3-*O*-rhamnoside	C_21_H_20_O_11_	447.0935, 301, 300, 271, 243, 227, 151
33	8.025	isorhamnetin 3,7-*O*-diglucoside	C_28_H_32_O_17_	639.2326, 447, 315, 137
34	8.068	kaempferol-3-*O*-pentoside isomer	C_20_H_18_O_10_	417.0751, 285, 284, 255, 227
35	8.314	kaempferol-3-*O*-pentoside isomer	C_20_H_18_O_10_	417.0835, 285, 284, 255, 227
36	8.518	kaempferol-3-*O*-rhamnoside	C_21_H_20_O_11_	431.0968, 285, 284, 255, 227
37	9.572	luteolin	C_15_H_10_O_6_	285.0373, 133
38	9.606	quercetin	C_15_H_10_O_7_	301.0351, 179, 151
39	10.291	pinocembrin-7-*O*-glucoside	C_21_H_22_O_9_	417.1170, 255, 151
40	10.308	pinocembrin	C_15_H_12_O_4_	255.0677, 213
41	10.416	2′,4′,6′-trihydroydi-hydrochalcon-4′-*O*-glucoside	C_21_H_24_O_9_	419.1353, 257, 213, 173, 125
42	10.625	pinocembrin-*O*-galloylglucoside	C_28_H_26_O_13_	569.1292, 313, 255, 169, 125
43	10.836	kaempferol	C_15_H_10_O_6_	285.0407
44	11.458	pinocembrin-*O*-galloylglucoside	C_28_H_26_O_13_	569.1292, 313, 255, 169, 125
45	11.606	pinocembrin-*O*-galloylglucoside	C_28_H_26_O_13_	569.1292, 313, 255, 169, 125
46	11.614	2′,4′,6′-trihydroydi-hydrochalcon-*O*-glucoside 2′,6′-dihydroydihydrochalcone-4′-*O*-(4″,6″-hexahydroxydiphenoyl)-β-glucoside	C_21_H_24_O_9_	419.1353, 257, 213, 173
47	11.95	pinocembrin-7-*O*-(4″,6″-(*S*)-hexahydroxydiphenoyl)-*β*-glucoside	C_35_H_28_O_17_	719.1229, 301, 275, 257, 229
48	12.07	apigenin	C_15_H_10_O_5_	269.0831
49	12.786	pinocembrin-7-*O*-(4″,6″-(*R*)-hexahydroxydiphenoyl)-*β*-glucoside	C_35_H_28_O_17_	719.1229, 301, 275, 257, 229
50	13.034	pinocembrin 7-*O*-(3″-galloyl-4″,6″-(*S*)-hexahydroxydiphenoyl)-*β*-D-glucose	C_42_H_32_O_21_	871.1351, 569, 301, 275, 255
51	13.955	pinocembrin 7-*O*-(3″-galloyl-4″,6″-(*R*)-hexahydroxydiphenoyl)-*β*-D-glucose	C_42_H_32_O_21_	871.1351, 569, 301, 275, 255
52	14.319	Thonningianin A	C_42_H_34_O_21_	873.1490, 571, 301, 275, 257
Hydrolyzed tannins				
53	0.843	galloylglucose 1	C_13_H_16_O_10_	331.0651, 271, 211, 169, 125
54	0.856	HHDP-glucose 1	C_20_H_18_O_14_	481.0610, 301, 275, 229
55	1.075	HHDP-glucose 2	C_20_H_18_O_14_	481.0610, 301, 275, 229
56	1.204	galloylglucose 2	C_13_H_16_O_10_	331.0651, 271, 211, 169, 125
57	1.256	HHDP-glucose 3	C_20_H_18_O_14_	481.0610, 301, 275, 229
58	2.259	galloylglucose 3	C_13_H_16_O_10_	331.0688, 169, 125
59	3.323	galloylglucose 4	C_13_H_16_O_10_	331.0688, 271, 211, 169, 125
60	3.648	digalloylglucose 1	C_20_H_20_O_14_	483.0798, 423, 331, 313, 271, 211, 169, 125
61	3.889	tetragalloylglucose	C_34_H_28_O_22_	787.1920, 625, 463, 301, 300, 299, 271
62	4.01	digalloylglucose 2	C_20_H_20_O_14_	483.0753, 423, 331, 313, 271, 211, 169, 125
63	4.19	digalloylglucose 3	C_20_H_20_O_14_	483.0798, 331, 313, 271, 211, 169, 125
64	4.325	galloyl-HHDP-glucose isomer 1	C_27_H_22_O_18_	633.0707, 463, 301, 275, 229
65	4.333	digalloylglucose 4	C_20_H_20_O_14_	483.0798, 331, 313, 271, 211, 169, 125
66	4.743	galloyl-HHDP-glucose isomer 2	C_27_H_22_O_18_	633.0707, 463, 301, 275, 229
67	5.543	trigalloyl-glucose isomer	C_27_H_24_O_18_	635.0870, 465, 313, 169, 125
68	5.626	digalloyl-HHDP-glucose	C_34_H_26_O_22_	785.0790, 301, 275
69	5.847	trigalloyl-glucose isomer 1	C_27_H_24_O_18_	635.0870, 483, 313, 271, 211, 169, 125
70	6.009	trigalloyl-glucose isomer 2	C_27_H_24_O_18_	635.0870, 483, 465, 313, 271, 211, 169, 125
71	6.543	trigalloyl-HHDP-glucose	C_41_H_30_O_26_	937.1021, 301, 275, 169
72	6.768	tetragalloylglucose 1	C_34_H_28_O_22_	787.1920, 635, 617, 465, 447, 313, 169, 125
73	6.864	tetragalloylglucose 2	C_34_H_28_O_22_	787.1000, 635, 617, 465, 447, 313, 169, 125
74	7.371	pentagalloylglucose 1	C_41_H_32_O_26_	939.1093, 769, 617, 447, 313, 169
75	7.685	pentagalloylglucose 2	C_41_H_32_O_26_	939.1155, 769, 617, 465, 313,1 69, 125
76	8.704	2,6-dihydroxyacetophenone-4-*O*-HHDP-glucose	C_27_H_20_O_18_	631.0937, 301, 229
Procyanidins				
77	4.099	Procyanidin B1	C_30_H_26_O_12_	577.1349, 451, 425, 407, 289, 245,161, 125
78	4.279	Procyanidin B3	C_30_H_26_O_12_	577.1399, 451, 425, 407, 289, 245, 161, 125
79	5.731	(epi)catechin–(epi)catechin-3-*O*-gallate 1	C_37_H_30_O_16_	729.1467, 577, 559, 425, 407, 289, 269, 169, 125
80	6.151	Procyanidin B4	C_30_H_26_O_12_	577.1300, 451, 425, 407, 289, 245, 161, 125
81	6.196	(epi)catechin–(epi)catechin-3-*O*-gallate 2	C_37_H_30_O_16_	729.1467, 577, 559, 425, 407, 289, 269, 169, 125
82	7.251	(epi)catechin–(epi)catechin-3-*O*-gallate 3	C_37_H_30_O_16_	729.1412, 577, 559, 425, 407, 289, 269, 169, 125
83	8.011	(epi)catechin–(epi)catechin-3-*O*-gallate 4	C_37_H_30_O_16_	729.1412, 577, 559, 425, 407, 289, 269, 169, 125
Others				
84	9.197	(4′E)-2,3′-dihydroxy-3-methoxy-6′-methanone-benzophenone-4-*O*-*β*-D-glucopyranoside	C_25_H_28_O_11_	503.1559, 341, 323, 308, 293, 284, 201, 157, 124
85	9.339	(4′E)-2,4-dihydroxy-3-methoxy-6′-methanone-benzophenone-3′-*O*-*β*-D-glucopyranoside	C_25_H_28_O_11_	503.1559, 341, 323, 308, 293, 201, 157, 124

**Table 2:** Total phenolic content (TPC), total flavonoid content (TFC), total proanthocyanidins content (TPAC) and total tannin content (TTC) of the different eluate fractions in *P. chinense* leaves.

Sample	Mass of Dried Residue (g)	TPC (mg GAE/g DW)	TFC (mg RE/g DW)	TAPC (mg CE/g DW)	TTC (mg TAE/g DW)
PC-20	0.47 ± 0.04 ^a^	418.45 ± 10.82 ^e^	197.56 ± 9.47 ^d^	84.95 ± 3.32 ^e^	10.61 ± 0.21 ^d^
PC-40	0.61 ± 0.06 ^ab^	398.32 ± 14.37 ^d^	227.55 ± 5.89 ^e^	69.51 ± 1.79 ^d^	7.15 ± 0.45 ^b^
PC-60	0.98 ± 0.00 ^b^	38.12 ± 1.30 ^a^	7.28 ± 0.33 ^a^	7.45 ± 0.83 ^a^	8.94 ± 0.78 ^c^
PC-80	0.32 ± 0.02 ^a^	158.88 ± 0.88 ^b^	45.25 ± 1.31 ^b^	16.51 ± 1.70 ^b^	3.97 ± 0.69 ^a^
PC	/	221.24 ± 4.17 ^c^	92.12 ± 5.17 ^c^	48.84 ± 3.66 ^c^	3.68 ± 0.51 ^a^

^a–e^ Data with different superscript lowercase letters in the same column were significantly different (*p* < 0.05); / means no data.

**Table 3:** The content of monomer components in the different eluate fractions in *P. chinense* leaves (mg/g).

Phenolic Compounds	PC-20	PC-40	PC-60	PC-80	PC
Gallic acid	4.29 ± 0.04 ^a^	/	/	/	4.53 ± 0.01 ^a^
Protocatechuic acid	0.68 ± 0.1	/	/	/	/
Catechin	59.90 ± 1.72 ^b^	/	/	/	4.43 ± 0.03 ^a^
Epicatechin	2.01 ± 0.15 ^a^	/	/	/	6.85 ± 0.87 ^b^
Rutin	0.26 ± 0.00 ^b^	1.57 ± 0.09 ^c^	/	/	0.15 ± 0.03 ^a^
Iisoquercitrin	0.43 ± 0.02 ^a^	5.29 ± 0.21 ^b^	/	/	0.46 ± 0.00 ^a^
Kaempferol 3-*O*-rutinoside	0.69 ± 0.07 ^a^	1.29 ± 0.22 ^b^	/	/	2.78 ± 0.02 ^c^
Astragalin	0.29 ± 0.02 ^a^	34.11 ± 0.76 ^c^	/	/	8.33 ± 0.06 ^b^
Afzelin	/	20.27 ± 0.11 ^c^	1.00 ± 0.05 ^a^	/	1.27 ± 0.20 ^b^
Pinocembrin 7-*O*-β-D-glucoside	1.70 ± 0.29 ^a^	2.65 ± 0.01 ^b^	6.89 ± 0.06 ^c^	/	2.48 ± 0.04 ^b^
Quercetin	/	1.26 ± 0.02	1.53 ± 0.19	/	/
Kaempferol	/	0.68 ± 0.03 ^a^	0.93 ± 0.04 ^b^	0.93 ± 0.04 ^b^	/
PGHG	/	8.61 ± 1.05 ^a^	110.23 ± 11.04 ^d^	87.59 ± 16.72 ^c^	15.93 ± 0.07 ^b^
Ponocembrin	/	/	2.17 ± 0.22 ^a^	5.80 ± 0.93 ^b^	80.00 ± 6.94 ^c^
Thonningianin A	1.81 ± 0.42 ^a^	60.57 ± 2.09 ^c^	657.47 ± 29.78 ^e^	273.00 ± 54.22 ^d^	14.05 ± 0.00 ^b^

^a–e^ Data with different superscript lowercase letters in the same row were significantly different (*p* < 0.05); / means no data.

## Data Availability

The original contributions presented in this study are included in the article/Supplementary Materials. Further inquiries can be directed to the corresponding author.

## Supplementary Materials

The following supporting information can be downloaded at https://www.mdpi.com/article/10.3390/antiox15060709/s1. Figure S1: UV chromatogram of the *P. chinense* leaf crude extract; Figure S2: The influence of different alcohol concentrations on cell survival rate; Figure S3: The influence of different sample concentrations on cell survival rate. Table S1: Pearson correlation coefficients between bioactive components and biological activities of four *P. chinense* fractions (PC 20, PC 40, PC 60, PC 80).
