# Supplementary material for: Macroporous Resin Purification of Phenolics from Penthorum chinense Leaves: Phenolic Identification, Composition Analysis, and Biological Activities
Source: Antioxidants (Basel). 2026 Jun 3;15(6):709. doi: 10.3390/antiox15060709 (PMC13295974; doi:10.3390/antiox15060709)
Supplement: Supplementary file 1 [file antioxidants-15-00709-s001.zip › antioxidants-4280999-supplementary.pdf]

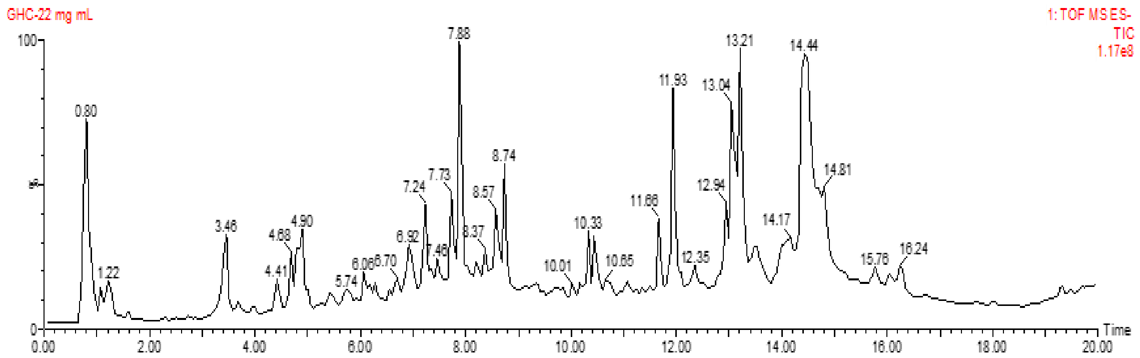

Figure S1. UV chromatogram of the *P. chinense* leaf crude extract.

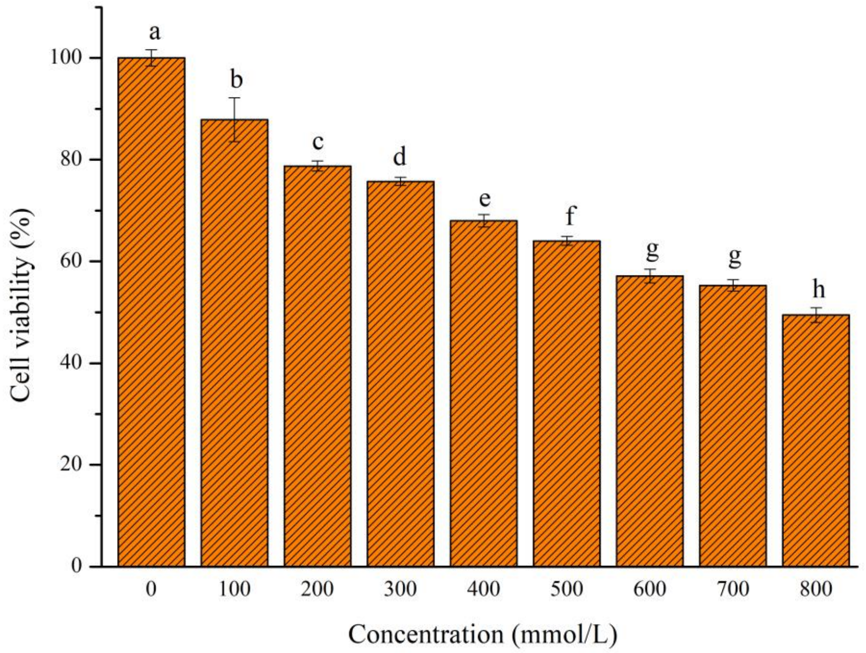

Figure S2. The influence of different alcohol concentrations on cell viability. Different lowercase letters indicate significant differences between the columns of the same color.

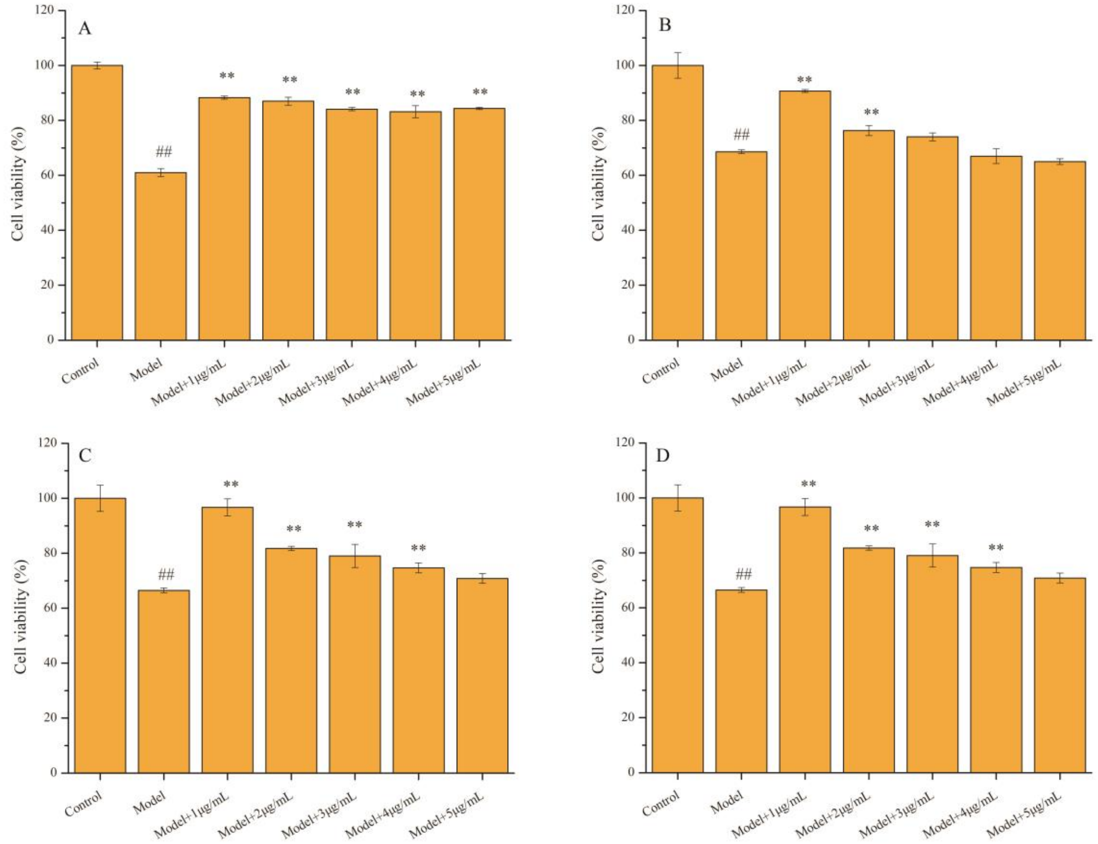

Figure S3. The influence of different concentrations of PC-20 (A), PC-40 (B), PC-60 (C) and PC-80 (D) on cell viability. Different lowercase letters indicate significant differences between the columns of the same color. Compared with the Control group, ## $P<0.01$; compared with the Model group, ** $P<0.01$.

Table S1. Pearson correlation coefficients between bioactive components and biological activities of four *Penthorum chinense* fractions (PC-20, PC-40, PC-60, PC-80)

	DPPH	ABTS	ADH	ALDH	α-glucosidase inhibition	pancreatic lipase inhibition	TPC	TFC	TPAC	TTC
DPPH	1	0.962**	-0.790*	-0.800**	-0.478	0.537	-0.812**	-0.723**	-0.697*	0.223
ABTS	0.962**	1	-0.868**	-0.864**	-0.546	0.670	-0.939**	-0.879**	-0.860**	0.000
ADH	-0.790*	-0.868**	1	0.992**	0.889**	-0.923**	0.882**	0.803*	0.905**	0.486
ALDH	-0.800**	-0.864**	0.992**	1	0.879**	-0.905**	0.862**	0.760**	0.872**	0.345
α-glucosidase inhibition	-0.478	-0.546	0.889**	0.879**	1	-0.933**	0.596*	0.464	0.682*	0.578*
pancreatic lipase inhibition	0.537	0.670	-0.923**	-0.905**	-0.933**	1	-0.775*	-0.716*	-0.853**	-0.722*
TPC	-0.812**	-0.939**	0.882**	0.862**	0.596*	-0.775*	1	0.977**	0.976**	0.302
TFC	-0.723**	-0.879**	0.803*	0.760**	0.464	-0.716*	0.977**	1	0.958**	0.321
TPAC	-0.697*	-0.860**	0.905**	0.872**	0.682*	-0.853**	0.976**	0.958**	1	0.485
TTC	0.223	0.000	0.486	0.345	0.578*	-0.722*	0.302	0.321	0.485	1

Note: **indicates the extremely significant correlation (P<0.01); *indicates the significant correlation (P<0.05).
